# Creating opportunities to improve detection of older adult abuse: a national interRAI study

**DOI:** 10.1186/s12877-022-02938-3

**Published:** 2022-03-17

**Authors:** Yvette Hall, Jim Smith, Robin M. Turner, Philippa Greco, Kenny Hau, Yoram Barak

**Affiliations:** 1 Department of Psychological Medicine, Dunedin School of Medicine, University of Otago, PO Box 56, Dunedin, New Zealand; 2 Biostatistics Centre, University of Otago, Dunedin, New Zealand; 3 Southern District Health Board, Dunedin, New Zealand

**Keywords:** Older adult abuse screening, InterRAI, Older adults, Abuse, Screening

## Abstract

Despite being recognized as a major global health issue, older adult abuse (OAA) remains largely undetected and under-reported. Most OAA assessment tools fail to capture true prevalence. Follow up of patients where abuse exposure is not easily determined is a necessity. The interRAI-HC (International Resident Assessment Instrument—Home Care) currently underestimates the extent of abuse. We investigated how to improve detection of OAA using the interRAI-HC. Analysis of 7 years of interRAI-HC data from an Aotearoa New Zealand cohort was completed. We identified that through altering the criteria for suspicion of OAA, capture rates of at-risk individuals could be nearly doubled from 2.6% to 4.8%. We propose that via adapting the interRAI-HC criteria to include the "unable to determine" whether abuse occurred (UDA) category, identification of OAA sufferers could be substantially improved. Improved identification will facilitate enhanced protection of this vulnerable population.

## Introduction

Older adult abuse (OAA) is now recognized as a major health issue in older populations worldwide. Despite this, effective strategies for the detection of OAA have been difficult to develop and implement. Consequently, the majority of sufferers remain unidentified and unprotected [1–3]. The World Health Organization (WHO) defines OAA as the mistreatment or neglect of persons aged 65 years or older, where this abuse, or neglect, may be either intentional or unintentional [4]. OAA can be a single incident, or repeated act, that causes harm or distress to an older adult, occurring within a relationship in which there is the expectation of trust between the abused and the perpetrator [5]. The impacts of OAA are severe in many cases and OAA is associated with increased rates of depression, anxiety, drug and alcohol abuse, chronic pain, as well as overall morbidity and mortality [5].

To accurately identify and protect sufferers of OAA, exact and extensive epidemiological data is required [4]. Current published data concerning OAA prevalence is extremely variable, with 0.8% to 40.4% of elders reporting OAA across Europe and the USA, with substantial variation by location and ethnicity [1, 6, 7]. Generally, an estimated overall prevalence of around 10% is currently accepted based on available evidence in New Zealand and worldwide; however, this is likely to be an underestimation of true OAA rates [8]. Regardless, only a small proportion of these individuals are known to protective or social services [9].

A panoptic view of tools for detecting OAA demonstrates a lack of adequate sensitivity and thus abuse identification is fraught with challenges in this vulnerable population [1]. Routine community-based screening would be a valuable first step towards improving elder abuse detection and response, however, more work is needed to identify a reliable screening tool that is acceptable to primary care practitioners and their patients. Further, prospective barriers and enablers to the use of such a screening tool require more extensive assessment [10]. The interRAI-HC (International Resident Assessment Instrument—Home Care) is an evidence-based, 236-item comprehensive clinical assessment for people with an age-related disability affecting their function, which is likely to continue for a minimum of 6 months and creates a potential need for support services. It is an assessment used for community dwelling adults, completed by certified nurses or social workers, and encompasses a wide range of aspects of an older person's life, including physical, psychological, and cognitive domains [11]. In Aotearoa New Zealand, the interRAI-HC is a mandatory component of a routine needs assessment, performed when older adults are evaluated for home support or transfer to a care facility. As such, it also offers a unique screening opportunity for OAA. Notably, the interRAI-HC assessment is also used internationally, offering broader prospective applications on a global scale [12]. Recently, we have demonstrated that the true prevalence of OAA is not comprehensively captured by the interRAI-HC [13].

Compared with other forms of interpersonal violence, OAA is under-researched [14]. Few protocols that have been trialed in “real-life” situations to identify victims of OAA. An Emergency Department protocol for identifying suspected OAA victims was tested retrospectively in 1988; however, the study lacked sufficient power to reach clinically meaningful conclusions [15]. Over twenty years later, several research groups trialed a range of protocols, including three different approaches: (1) telemedicine; (2) an observation-based Emergency Department screening tool; and (3) the use of electronic health records to facilitate elder mistreatment screening [16–18]. These trials, however, are of limited application in practice, as they were all completed only within emergency care settings and were geared towards identification of severe cases of active suffering, rather than prevention. Each of these approaches relies on clinicians’ adapting novel methodology to actively pursue and identify OAA victims, whereas accessible datasets such as the interRAI offer standardized, clinically oriented assessments of older adults and are less reliant on opportunistic screening.

A major pitfall of the interRAI assessment in detecting OAA is that the current threshold to trigger a comprehensive abuse assessment, termed the abuse clinical assessment protocol (A-CAP), leaves room for error. Indeed, critical abuse screening questions are asked in such a manner that individuals can state that they are “unable to determine” (UDA) whether abuse has occurred. Given the nature of these questions, we hypothesize that respondents selecting this option would be at higher risk of having experienced abuse, further compounding the underdetection of vulnerable individuals.

The aim of the present study is to use a retrospective analysis of routinely collected data from the interRAI-HC assessment for triggering abuse assessments in an Aotearoa New Zealand cohort to test the hypothesize that OAA capture rates could be substantially improved through deliberate modification of selected criteria. Further, we discuss the major challenges of using the interRAI for OAA detection in practice and prospective implications of using a standardized tool for identifying and preventing OAA.

## Methods

### Participants

Participants were New Zealanders aged 65 years and older who completed their first InterRAI-HC assessment between January 2013 and August 2019, within the Southern District Health Board (SDHB) region, Aotearoa New Zealand. If participants underwent multiple assessments, only data from the initial assessment was reviewed. Participant data was completely anonymized. Participant age was recorded at the time of assessment. Only InterRAI-HC data from participants consenting for use in research (96%) was included, as per previous protocols for using interRAI-HC data [19]. During the study period, 19,594 interRAI-HC first assessments were undertaken in the SDHB. There were no changes in the interRAI protocol during the study period.

Of the 19,594 interRAI-HC first assessments undertaken during the study period, 710 participants were excluded from the final analysis, including 693 interviewees younger than 65-years-old and 17 interviewees who either were in a coma or were recorded as having "null" information relevant to this analysis.

### Data analysis

This study compared the characteristics of the general population undergoing interRAI assessment to those considered at high risk of abuse who triggered the A-CAP. In addition, characteristics of the general population and those at high risk were compared to those for whom the risk of abuse was considered “unable to be determined” (UDA). We hypothesized the UDA group will also be at-risk for OAA. For the purposes of our analysis, the UDA group included any participant who did not trigger the A-CAP, but did select “unable to be determined” as the answer to one or more of the screening questions listed in Table 1. Exact criteria for triggering the A-CAP are also detailed in Table 1.

**Table 1:** Questions that contribute to the abuse CAP (A-CAP) group

Abusive Relationship			
Question	Trigger Value	Actual Value	Triggered
[Level 1] MODERATE RISK STATUS persons who have one or more of the following direct indicators of abuse are present			
[F1e] Fearful of family member	2,3,4…	Not Answered	
[F1f] Neglected or abused	2,3,4…	Not Answered	
[J2t] Poor hygiene	2,3,4…	Not Answered	
and less than 2 of the following “stressors” are present			
[E1i] Withdrawal	1,2,3…	Not Answered	
[E1j] Reduced social interactions	1,2,3…	Not Answered	
[F2] Lonely	1…	Not Answered	
[K2a] Weight loss	1…	Not Answered	
[K2c] Fluid intake	1…	Not Answered	
[sBMI] Body Mass Index Scale	1—18…	0	
[J6a] Unstable conditions	1…	Not Answered	
[J7] Self-rated health	3…	Not Answered	
[M3] Drug adherence	1,2…	Not Answered	
[A13c] Better Living Elsewhere	1…	Not Answered	
[F1d] Openly expresses conflict w/family	2,3,4…	Not Answered	
[P2b] Informal helper stress	1…	Not Answered	
[sDRS] Depression Rating Scale indicates a depressive disorder	3—14…	0	
[Level 2] HIGHEST RISK STATUS triggered includes persons who have one or more of the following direct indicators of abuse are present			
[F1e] Fearful of family member	2,3,4…	Not Answered	
[F1f] Neglected or abused	2,3,4…	Not Answered	
[J2t] Poor hygiene	2,3,4…	Not Answered	
and Two or more of the following “stressors” are present			
[E1i] Withdrawal	1,2,3…	Not Answered	
[E1j] Reduced social interactions	1,2,3…	Not Answered	
[F2] Lonely	1…	Not Answered	
[K2a] Weight loss	1…	Not Answered	
[K2c] Fluid intake	1…	Not Answered	
[sBMI] Body Mass Index Scale	1—18…	0	
[J6a] Unstable conditions	1…	Not Answered	
[J7] Self-rated health	3…	Not Answered	
[M3] Drug adherence	1,2…	Not Answered	
[A13c] Better Living Elsewhere	1…	Not Answered	
[F1d] Openly expresses conflict w/family	2,3,4…	Not Answered	
[P2b] Informal helper stress	1…	Not Answered	
[sDRS] Depression Rating Scale indicates a depressive disorder	3—14…	0	

Trigger Value = 0-never; 1-more than 30 days ago; 2–8 to 30 days ago; 3–4 to 7 days ago; 4-in the last 3 days; 8-unable to determine

Actual Value = value assigned by the assessor

Triggered = the summary computerized algorithm indicating “yes” or “no” for CAP triggering

Five items from the interRAI-HC dataset were analyzed for each of the comparison groups: age; gender; living situation; depression; and independence in daily decision-making. These items were previously shown by our group to be associated with abuse potential [13]. Our primary definition of depression was a Depression Rating Scale (DRS) score of ≥ 3, as this is considered to reflect clinically meaningful depression [20, 21]. The interRAI-HC assesses the ability of the participant to make decisions regarding tasks of daily life, stratified into six categories as per Table 2. With respect to decision-making capacity, we collated participants with “independent” or “modified independence” into a single group termed “Independent” in decision-making. We considered participants in all other categories to have “Impaired” decision-making capabilities.

**Table 2:** *(percent of the total sample)

Characteristic	Total Sample	%	A-CAP	%	UDA	%	General sample (non A-CAP & non UDA)	%	A-CAP vs. UDA	General sample vs UDA
									*P*-Value	*P*-Value
Number of people	18,884	100	493	*2.6	414	*2.2	17,977	*95.2		
Gender									0.011	0.108
Female	11,110	58.8	231	46.9	229	55.3	10,650	59.2		
Male	7774	41.2	262	53.1	185	44.7	7327	40.8		
Age (years)									0.431	< 0.001
Total population (mean)	82.6		80		80.4		82.7			
Female (mean)	83.2		80.7		81.5		83.3			
Male (mean)	81.8		79.3		79.1		81.9			
Range	65–109		65–101		65–99		65–109			
Living situation									< 0.001	< 0.001
Alone	9838	52.1	274	55.6	179	43.2	9385	52.2		
With someone else	9046	47.9	219	44.4	235	56.8	8592	47.8		
Depression									0.500	< 0.001
Depressed (DRS ≥ 3)	3496	18.5	147	29.8	115	27.8	3234	18.0		
Not depressed (DRS 0–2)	15,388	81.5	346	70.2	299	72.2	14,743	82.0		
Decision making									0.953	< 0.001
Independent	10,084	53.4	139	28.2	116	28.0	9829	54.7		

### Statistical analyses

Descriptive statistics are presented for all variables, including demographics and health variables. The χ^2^ test was used to compare the distribution of categorical variables between the A-CAP, UDA and general (non A-CAP non UDA) groups. Missing data was minimal (< 0.07% per variable). Student’s *t*-test was used to compare age between groups.. All available data were used for each statistical test. Analyses were conducted in Stata, version 15.1 (StataCorp).

### Ethics

Ethical approval (HD17/064) was obtained from the University of Otago Ethics Committee and the Department of Psychological Medicine Ethics Committee.

## Results

### Participants

Of the 19,594 interRAI-HC first assessments undertaken during the study period, 710 participants were excluded from the final analysis, including 693 interviewees younger than 65-years-old and 17 interviewees who either were in a coma or were recorded as having "null" information relevant to this analysis. Data from 18,884 participants (mean age 82.6 years; age range, 65–109 years; 11,110 (59%) female and 7,774 (41%) male) were included in the final analysis.

### The general cohort, A-CAP and UDA groups

Each of our five variables were compared between the A-CAP and UDA groups, as detailed in Table 2. Within the total elderly sample (*n* = 18,884; mean age 82.6 [7.5] years), 493 interviewees (2.6%) triggered the A-CAP versus 414 (2.2%) interviewees who met criteria for the UDA category.

The mean age [± SD] of A-CAP interviewees was 80.0 [7.7] years (range: 65–101). Similarly, the UDA category of interviewees had a mean [± SD] age of 80.4 [7.5] years (range: 65–99). There was no difference in mean age between these two groups (*p* = 0.43).

There was a gender difference between the A-CAP and UDA groups, with 53.1% versus 44.7% male gender, respectively (*p* = 0.011). No difference in gender was identified between the UDA group and general sample (*p* = 0.108).

With respect to the impacts of living situation, respondents for whom their responses triggered the A-CAP were more likely to be living alone (55.6%, *n* = 274) compared to those in the UDA group (43.2%, *n* = 174) (*p* < 0.001). Interestingly, a similar difference was also observed between the general cohort and UDA group, with 52.2% of all participants reported to be living alone, (*p* < 0.001).

There was no difference in depression status (29.8% vs 27.8%;, *p* = 0.500), or independence in decision-making (28.2% vs 28.0%;, *p* = 0.953) between the A-CAP and UDA groups, respectively. However, the general cohort reported lower rates of depression (18.5%,, *p* < 0.001) and greater independence in decision-making (53.4%,, *p* < 0.001) than the UDA cohort.

## Discussion

OAA has a profound impact on physical and psychosocial health [10]. Despite this, many countries have only minimal prevalence or incidence data available for OAA [8]. Reported OAA rates are thought to be a substantial underestimation because of a reluctance to report abuse and only an estimated 20% of victims are known to protection services [9, 22]. It is clear that a valid and reliable screening instrument for OAA detection is required, though such a tool has proven challenging to develop [1, 23]. Effective screening and detection of OAA would be invaluable in victim protection and implementing prevention strategies.

The interRAI-HC has the potential to provide one such mechanism of abuse detection. The interRAI was designed to highlight opportunities for health improvement as well as any obvious risks to a person's health. In this study, we evaluated participants who undertook the interRAI-HC assessment in the SDHB of Aotearoa New Zealand and hypothesized that the inclusion of the UDA group could improve OAA capture rates and identify those at increased risk of OAA. As compared to the general cohort of elderly participants, our analysis demonstrates that the UDA group more closely resembles the group which trigger the interRAI-HC A-CAP across a number of important variables.

Significant differences were identified between groups for both age and gender, however, the practical significance of these differences are unclear. In terms of gender distribution, the UDA group (44.7% male) was in fact more similar to the general population (41.2% male) than those who triggered the A-CAP (53.1% male), which may reflect a reluctance amongst females to directly report abuse or major abuse risk factors. With respect to age, there was no difference between the UDA and A-CAP groups (80.4 and 80.0 years old, respectively), but UDA was significantly different from the general population (82.6 years old). The age ranges of each group, however, was very similar and it would seem unlikely that a mean age difference of 2.2 years holds much clinical significance in this context.

Non-disclosure of abuse remains a core issue in OAA underdetection and research indicates a significant risk factor for abuse is sharing a living environment and that the perpetrator of OAA is frequently living with the victim [8]. Interestingly, in our analysis, the proportion of interviewees who lived alone was significantly lower in the UDA group compared with the general population. This may reflect that even though living with others is a known risk factor for experiencing OAA, those who live with others may be less likely to actually report abuse, possibly due to fear of the social implications that this may have. Furthermore, due to socioeconomic status and health constraints in Aotearoa New Zealand, interviews are often conducted in group settings where privacy may be compromised [4]. Older adults interviewed with other people present have different patterns of reporting, often with differing levels of disclosure than when interviewed alone. People in groups tend to retell events that have motives relating to the needs of the group dynamics rather than providing a strictly factual report [24].

Depression and impaired decision making capacity emerged from our analysis as factors which were remarkably consistent between the A-CAP and UDA groups, whilst significantly different from the general cohort. Interviewees who were depressed were overrepresented in the A-CAP (29.8%) and UDA (27.8%) groups versus the general cohort (18.5%). Similarly, 53.4% of the general cohort were independent in their decision-making capacity, compared to only 28.2% and 28.0% of the A-CAP and UDA groups, respectively. Depression and decision making capacity are, therefore, important variables captured by the interRAI-HC, correlated with at-risk status for OAA in our cohort. Indeed, depression and mental illness have been previously identified as common sequelae of OAA, which is likely reflected in our cohort [8]. Furthermore, functional impairment is a well-documented independent risk factor for experiencing OAA and those with impaired decision-making are an inherently more vulnerable population, at risk of exploitation and abuse [8]. Together, these data suggest that, alongside inclusion of the UDA group, there may be value in altering the interpretation of the interRAI-HC depression and independence in decision making variables to increase the sensitivity of identifying individuals at-risk of OAA.

A variety of factors makes the screening for OAA challenging [10]. Mistreatment may occur as a single act or as a chronic, subtle series of events. Further, expectations across different settings may also influence the identification and definition of cases [5]. The link between OAA and child abuse is complex but potentially relevant to the dynamics underlying reluctance to disclose abuse. An abusing parent may, in ageing, become a victim of OAA – with the now-adult child becoming the caretaker as well as the OAA perpetrator [25, 26]. Family norms contribute to abuse tolerance, for instance, childhood experience of abuse. Neglect or violence normalizes these behaviours and can be a barrier in the disclosure of intrafamilial violence. At any point in the family history, the "trusting relationship" is evolving and cannot be taken for granted especially with lifelong violent family relationships, where at times the relationship is based on fear and intimidation [27]. Reluctance to report OAA could mean that older adults who are more disabled, isolated, and dependent are more at risk of OAA, as our results suggest. Moreover, vulnerable OAA victims will often tolerate abusive behaviours to maintain the benefits and contact or support available, in parallel to the abusive relationship. Additionally, coping strategies of denial, minimization, low self-esteem and dependence can all become barriers to reporting of abuse cases [28]. Finally, self-neglect, a significant area of concern among older adults with respect to their health and safety is often confounded with abuse. Current OAA screening tools are limited in their ability to distinguish between elders who suffer self-neglect and those who are maltreated or neglected by those charged with caring for them. However, the interRAI-HC A-CAP is specifically designed to assess relational aspects of OAA and does not explicitly screen for behaviours such as self-neglect [29]. Consideration of each of these variables, within the framework of the interRAI-HC assessment, would be valuable in appropriately gearing this tool towards effective OAA detection.

Several population factors are relevant for repurposing the interRAI-HC assessment both in Aotearoa New Zealand and overseas. In Aotearoa New Zealand, individuals completing the interRAI-HC assessment are older and inherently more frail than the general older adult population [19]. The results of this study may, therefore, not be completely generalizable to the general older adult population; however, they are directly relevant to individuals undergoing the interRAI-HC assessment for any reason. Many screening tools including the interRAI-HC rely on self-reporting and, in Aotearoa New Zealand, older adults culturally tend towards non-disclosure and stoicism—both traits likely to contribute to cloaking the topic of abuse [30]. Furthermore, stigma is a potent barrier to seeking help for mental and physical health problems and is a particularly significant issue embedded in the culture of small rural areas of Aotearoa New Zealand [31, 32].

Assessor variability is also an important consideration in interpreting interRAI-HC abuse assessments. When an interRAI-HC assessor cannot conclusively identify the presence or absence of OAA, they report that they are unable to determine if abuse has occurred. Colloquially, we refer to having a “gut feeling” when unable to base decisions on facts alone. The importance of “gut feeling” is difficult to quantify, but we rely on it in our professional roles to detect situations where a patient is at risk of a negative outcome and this is no different in OAA [33]. “We've heard reports oftentimes, family members who come into the bank with grandma and they can tell the person is pushy…and they have a 'gut feeling' something is going on that shouldn't be” [34]. Ensuring that assessment tools are as objective and accurate as possible is essential to maximize the detection of at-risk individuals, as is the appropriate acknowledgement of “gut feeling” responses in face-to-face assessment tools, where a low threshold for more comprehensive abuse assessment should be commonplace.

Ironically, the absence of accurate OAA prevalence data itself remains a major limitation to developing accurate detection tools. Similar to other studies which investigate tools for OAA detection, our work assesses the important factors that place an individual at risk of experiencing OAA. Unfortunately, without access to true prevalence data, the findings of such studies can only be correlative in nature, providing a best approximation of which individuals require comprehensive assessment for OAA. We acknowledge that our work provides only a preliminary analysis of the interRAI-HC assessment as a potentially effective tool for OAA. However, our findings identify depression and independence in decision making as prospective risk factors for experiencing OAA and provide support for triggering the A-CAP for individuals placed in the UDA group.

Routine, community-based screening would be a valuable first step towards improving OAA detection and response internationally, however, more research is required to develop a reliable screening system. We propose that interRAI-HC could become such a tool with an appropriate adaptation of A-CAP triggering and, in the first instance, we suggest that more comprehensive abuse assessment of participants placed in the UDA category could meaningfully improve interRAI-HC-based detection of at-risk individuals for OAA.

## Data Availability

The data analyzed in this study is freely available by request to the corresponding author. Data will be provided as a pdf or word document.

## Abbreviations

interRAI-HC: International Resident Assessment Instrument—Home Care
A-CAP: Abuse Clinical Assessment Protocol
OAA: Older Adult Abuse
UDA: Unable to Determine

## References

[1] De Donder L, Luoma ML, Penhale B, Lang G, Santos AJ, Tamutiene I, Koivusilta M, Schopf A, Ferreira Alves J, Reingarde J (2011). European map of prevalence rates of elder abuse and its impact for future research. Eur J Ageing.

[2] Beach SR, Carpenter CR, Rosen T, Sharps P, Gelles R (2016). Screening and detection of elder abuse: Research opportunities and lessons learned from emergency geriatric care, intimate partner violence, and child abuse. J Elder Abuse Negl.

[3] Rohringer TJ, Rosen TE, Lee MR, Sagar P, Murphy KJ (2020). Can diagnostic imaging help improve elder abuse detection?. Br J Radiol.

[4] Peri K, Fanslow J, Hand J, Parsons J (2009). Keeping older people safe by preventing elder abuse and neglect. Soc Policy J N Z.

[5] WHO: Missing voices: views of older persons on elder abuse. In*.*: WHO; 2002.

[6] DeLiema M, Gassoumis ZD, Homeier DC, Wilber KH (2012). Determining prevalence and correlates of elder abuse using promotores: low-income immigrant Latinos report high rates of abuse and neglect. J Anim Physiol Nutr.

[7] Eslami B, Di Rosa M, Barros H, Stankunas M, Torres-Gonzalez F, Ioannidi-Kapolou E, Lindert J, Melchiorre MG (2017). Lifetime abuse and perceived social support among the elderly: a study from seven European countries. Eur J Pub Health.

[8] Lachs MS, Pillemer KA (2015). Elder Abuse. N Engl J Med.

[9] Cooper C, Selwood A, Livingston G (2008). The prevalence of elder abuse and neglect: a systematic review. Age Ageing.

[10] Ries NM, Mansfield E (2018). Elder abuse: The role of general practitioners in community-based screening and multidisciplinary action. Australian Journal of General Practice.

[11] Barak Y, Leitch S, Glue P: The Great Escape. Centenarian Escapers’ exceptional health: it’s the journey that counts. Under Submission 2019:1–13.

[12] Salahudeen MS, Nishtala PS (2019). A Systematic Review Evaluating the Use of the interRAI Home Care Instrument in Research for Older People. Clin Gerontol.

[13] Hall YS, Barak Y, Greco P, Hau K: Older Adults Abuse: Analysis of a New Zealand national dataset. International Psychogeriatrics 2020. 10.1017/S1041610220001520 32830636

[14] Cooper C, Livingston G (2016). Intervening to reduce elder abuse: challenges for research. Age Ageing.

[15] Jones J, Dougherty J, Schelble D, Cunningham W (1988). Emergency department protocol for the diagnosis and evaluation of geriatric abuse. Ann Emerg Med.

[16] Bloemen EM, Rosen T, Cline Schiroo JA, Clark S, Mulcare MR, Stern ME, Mysliwiec R, Flomenbaum NE, Lachs MS, Hargarten S (2016). Photographing Injuries in the Acute Care Setting: Development and Evaluation of a Standardized Protocol for Research, Forensics, and Clinical Practice. Acad Emerg Med.

[17] Cannell B, Weitlauf J, Livingston MD, Burnett J, Parayil M, Reingle Gonzalez J (2020). Validation of the detection of elder abuse through emergency care technicians (DETECT) screening tool: a study protocol. BMJ open.

[18] Rosen T, Platts-Mills TF, Fulmer T (2020). Screening for elder mistreatment in emergency departments: current progress and recommendations for next steps. J Elder Abuse Negl.

[19] Leitch S, Glue P, Gray AR, Greco P, Barak Y (2018). Comparison of Psychosocial Variables Associated With Loneliness in Centenarian vs Elderly Populations in New Zealand. JAMA Netw Open.

[20] Huang Y, Carpenter I (2011). Identifying elderly depression using the Depression Rating Scale as part of comprehensive standardised care assessment in nursing homes. Aging Ment Health.

[21] Penny K, Barron A, Higgins A-M, Gee S, Croucher M, Cheung G (2016). Convergent Validity, Concurrent Validity, and Diagnostic Accuracy of the interRAI Depression Rating Scale. J Geriatr Psychiatry Neurol.

[22] Oosterlee A, Vink RM, Smit F (2009). Prevalence of family violence in adults and children: estimates using the capture-recapture method. Eur J Pub Health.

[23] Fulmer T, Guadagno L, Bitondo Dyer C, Connolly MT (2004). Progress in elder abuse screening and assessment instruments. Journal of the American Geriatric Society.

[24] Vernham Z, Granhag P, Mac GE: Detecting Deception within Small Groups: A Literature Review. Frontiers in psychology 2016, 7(1012). 10.3389/fpsyg.2016.01012 PMC4927566 27445957

[25] Li G, Dong  X (2016). Intergenerational Transmission of Violence: The Association between Child Abuse and Elder abuse. The Gerontologist.

[26] Utech MR, Garrett RR (1992). Elder and Child Abuse: Conceptual and Perceptual Parallels. J Interpers Violence.

[27] Johnson  LA (1995). Spurious assumptions about ED domestic violence victim caseloads. Ann Emerg Med.

[28] National Research Council (2003). Elder Mistreatment: Abuse, Neglect, and Exploitation in an Aging America.

[29] Betini RSD, Hirdes JP, Lero DS, Cadell S, Poss J, Heckman G (2017). A longitudinal study looking at and beyond care recipient health as a predictor of long term care home admission. BMC Health Serv Res.

[30] Braun V (2008). "She'll be right"? National identity explanations for poor sexual health statistics in Aotearoa/New Zealand.. Social science & medicine.

[31] Judd F, Jackson H, Komiti A, Murray G, Fraser C, Grieve A, Gomez R (2006). Help-seeking by rural residents for mental health problems: the importance of agrarian values. Aust N Z J Psychiatry.

[32] StatsNZ: Population ageing in New Zealand.Key Statistics2000:7.

[33] Agnew T (2006). Beating abuse. Nurs Older People.

[34] Bompey N: Elder abuse, exploitation rising. In:Asheville Citizen - Times. Asheville, N.C.; 2011.

